# Comparative transcriptome analysis of the floral transition in *Rosa chinensis* ‘Old Blush’ and *R*. *odorata* var. *gigantea*

**DOI:** 10.1038/s41598-017-05850-8

**Published:** 2017-07-20

**Authors:** Xuelian Guo, Chao Yu, Le Luo, Huihua Wan, Yushu Li, Jia Wang, Tangren Cheng, Huitang Pan, Qixiang Zhang

**Affiliations:** Beijing Key Laboratory of Ornamental Plants Germplasm Innovation & Molecular Breeding, National Engineering Research Center for Floriculture, Beijing Laboratory of Urban and Rural Ecological Environment, Key Laboratory of Genetics and Breeding in Forest Trees and Ornamental Plants of Ministry of Education, School of Landscape Architecture, Beijing Forestry University, Beijing, 100083 China

## Abstract

The floral transition is a crucial developmental event, but little is known about the underlying regulatory networks in seasonally and continuously flowering roses. In this study, we compared the genetic basis of flowering in two rose species, *Rosa chinensis* ‘Old Blush’, which flowers continuously, and *R*. *odorata* var. *gigantea*, which blooms in early spring. Gene ontology (GO) terms related to methylation, light reaction, and starch metabolism were enriched in *R*. *odorata* var. *gigantea* and terms associated with sugar metabolism were enriched in *R*. *chinensis* ‘Old Blush’ during the floral transition. A MapMan analysis revealed that genes involved in hormone signaling mediate the floral transition in both taxa. Furthermore, differentially expressed genes (DEGs) involved in vernalization, photoperiod, gibberellin (GA), and starch metabolism pathways converged on integrators, e.g., *LFY*, *AGL24*, *SOC1*, *CAL*, and *COLs*, to regulate the floral transition in *R*. *odorata* var. *gigantea*, while DEGs related to photoperiod, sugar metabolism, and GA pathways, including *COL16*, *LFY*, *AGL11*, *6PGDH*, *GASA4*, and *BAM*, modulated the floral transition in *R*. *chinensis* ‘Old Blush.’ Our analysis of the genes underlying the floral transition in roses with different patterns of flowering provides a basis for further functional studies.

## Introduction

The floral transition is an important developmental event orchestrated by external and developmental stimuli; for example, photoperiod, vernalization, autonomous, aging, gibberellin (GA), and sugar metabolism pathways^1, 2^ interact to activate or inhibit the floral transition via a small set of floral integrators, including *FLOWERING LOCUS T* (*FT*), *CONSTANS-LIKE* (*COL*), *SUPPRESSOR OF OVEREXPRESSION OF CO1* (*SOC1*), *FLOWERING LOCUS C* (*FLC*), and floral meristem identity genes, e.g., *LEAFY* (*LFY*), *APETALA1* (*AP1*), *AGAMOUS-LIKE* (*AGL*), *CAULIFLOWER* (*CAL*), and *SEPALLATA* (*SEP*).

Considerable progress has been made toward identifying the molecular networks underlying the floral transition in the annual plant *Arabidopsis thaliana* ^3^. However, relatively little is known about the regulation of the floral transition in perennial plants. In perennials, only a portion of buds have competence to complete the phase transition from vegetative to reproductive growth in inductive conditions, while others remain in vegetative growth, indicating the ability to revert back from reproductive to vegetative growth. In temperate regions, most perennials require a period of winter cold (vernalization) to induce the floral transition and show seasonal flowering. However, a small number of perennials, such as *Rosa chinensis* and *Fragaria vesca*, can flower continuously in advantageous environmental conditions. The duration of flowering is crucial for the value of ornamental plants, such as modern roses and chrysanthemums.

Some progress has been made in understanding flowering perennials. Vernalization and sugar metabolism, which interacts with ABA signaling and aging pathways, are key factors inducing the floral transition in *Malus* × *domestica* ^4, 5^. The photoperiod-mediated *CO*/*FT* modules function as activators in the induction of the floral transition in chrysanthemums and poplars^6, 7^. Different from other species in the genus *Camellia*, the floral transition in *C*. *azalea* occurs in the spring; it is regulated by low temperatures, photoperiod, and hormone signals^8^. Jameson and Clemens have preliminarily determined inductive and non-inductive factors that influence the floral transition in New Zealand flora, including *Sophora*, *Clianthus*, and *Metrosideros* ^9^. The floral transition in *Metrosideros* is controlled by cool temperatures and photoperiod^10^, while floral initiation in *Sophora* does not respond to short day length, but is activated by vernalization^11^, and inflorescence initiation in *Clianthus* occurs in late autumn or early winter^12^.

Unlike the examples mentioned above, some perennial plants can transform between vegetative and reproductive growth throughout the year. For example, the floral transition in *Arabis alpina* is regulated by *PERPETUAL FLOWERING 1* (*PEP1*), an ortholog of *A*. *thaliana FLC*, which encodes a MADS-box transcription factor that inhibits the floral transition before the winter; the species can return to vegetative growth after flowering. Unexpectedly, mutations in *PEP1* enable *A*. *alpina* to flower continuously, without vernalization, indicating that the floral transition in *A*. *alpina* mainly depends on *PEP1* ^13^. It is noteworthy that the repression of vernalization on *PEP1* is only transient, and *PEP1* expression increases in warm temperatures^13^. However, the chromatin modification of *FLC* is stable, even if *A*. *thaliana* returns to warm temperatures^14^. This property distinguishes *A*. *alpina* from annual plants. The expression of *TFL1* inhibits the conversion from vegetative to reproductive growth in *A*. *thaliana* ^2^. Orthologs of *A*. *thaliana TFL1* have been identified in *Fragaria vesca* and continuous flowering (CF) roses^15^. The strawberry genome contains a 2-bp deletion in the first exon of *TFL1*, which contributes to CF. Likewise, the expression of *TFL1* is inhibited by a retrotransposon in CF roses^15^. GA plays an activating role in regulating the floral transition in *A*. *thaliana*, but GAs are maintained at low levels during the floral transition process in both CF and seasonal roses (OF), and increase after flowering^16^. Spraying application of GA_3_ could block the floral transition in OF roses, but not in CF roses. Moreover, paclobutrazol treatment does not induce secondary floral transition in OF roses. Thus, the floral transition in OF roses in the spring, but not in CF roses, is influenced by GA_3_^15^. In addition, rose *RoKSN* acts antagonistically with *RoFT*, competing with the bZIP transcription factor *RoFD* to inhibit or activate the floral transition^17^. Paradoxically, the floral transition in *Rosa rugosa* ‘Hamanasu’, a CF rose, is not controlled by the *KSN* ^*copia*^ allele^18^. Wang *et al*. proposed that CF is not a strictly qualitative trait, but a qualitative-quantitative trait^19^. These results indicate that floral genes other than *RoKSN* regulate the rose floral transition.

*R*. *chinensis* ‘Old Blush’ and *R*. *odorata* var. *gigantea* are diploid perennials, belonging to section *Chinenses* DC, from which modern roses, which share CF and tea scent, are derived. For simplicity, *R*. *chinensis* ‘Old Blush’ and *R*. *odorata* var. *gigantea* are abbreviated OB and GIG, respectively, throughout the text. OB flowers continuously in favorable temperature and photoperiod conditions, while GIG only blooms in the early spring. Studies of *A*. *thaliana* and other species have demonstrated that most flower-related genes are conserved across plant species^2, 20^. Thus, based on the complex integrated gene networks underpinning floral transition summarized in previous studies^20, 21^, a longitudinal comparative transcriptome analysis was performed during the floral transition process, which was divided into vegetative meristem (VM), pre-floral meristem (TM), and floral meristem (FM) stages. Differentially expressed genes (DEGs) between stages in each floral pathway were identified, and further interspecific comparative analyses were performed to investigate key candidate floral genes underlying the floral transition in the two species. This research provides valuable information on the physiology and molecular regulatory mechanisms of the floral transition in two roses that differ in flowering behavior.

## Results

### GO term enrichment in pairwise comparisons

To discover molecular changes during the floral transition in OB and GIG, floral gene expression was examined at the VM, TM, and FM stages (Supplementary Fig. S1). Moreover, secondary axillary buds that are unable to flower were also examined in GIG (referred to as SVM-GIG). A histological analysis indicated that they were in vegetative growth (Supplementary Fig. S1b).

**Figure 1:**
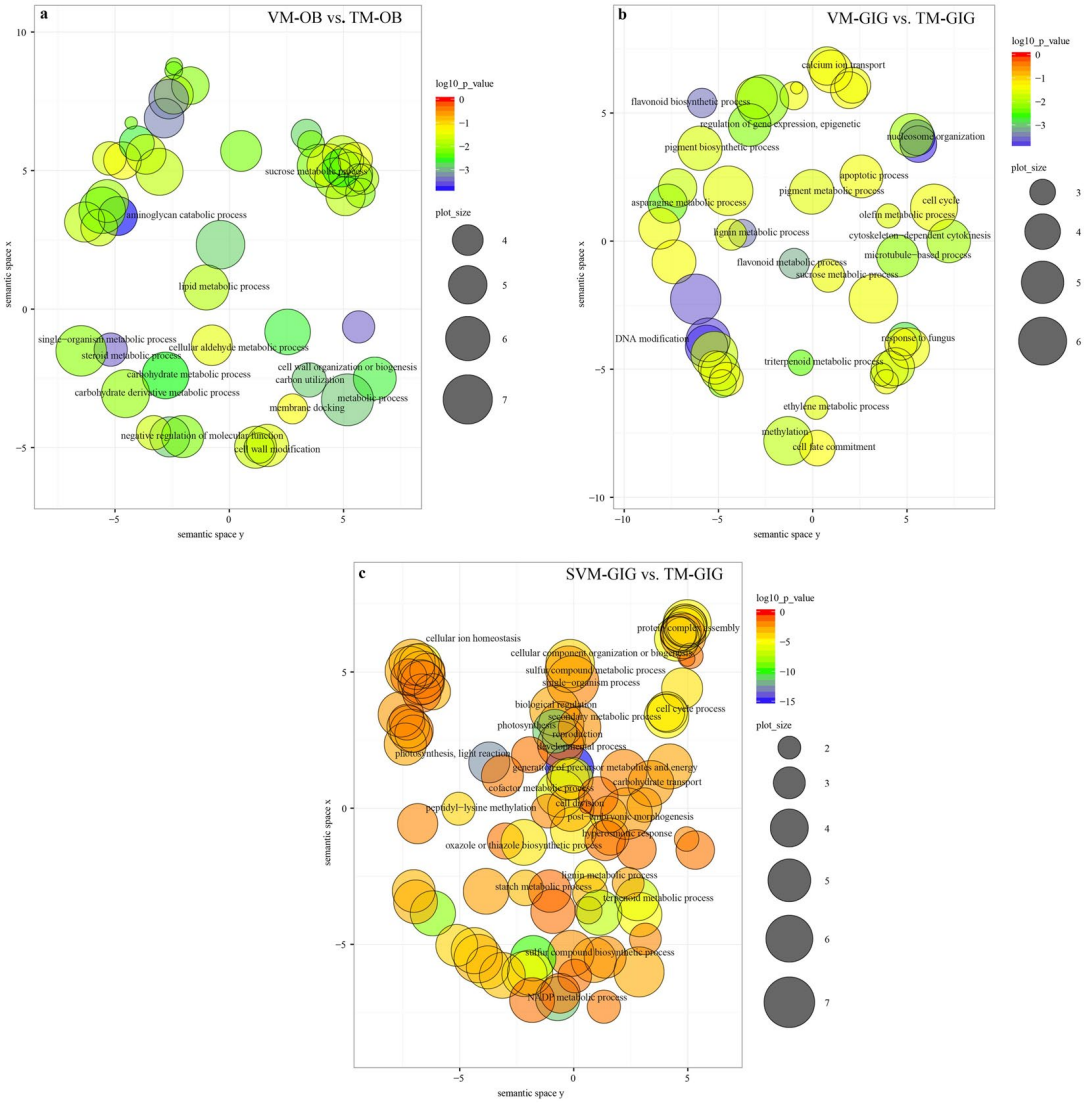
Scatterplot of enriched GO terms in pairwise comparisons. The scatterplot of enriched GO terms (p < 0.05) in biological process in the VM-OB vs. TM-OB comparison (**a**), in the VM-GIG vs. TM-GIG comparison (**b**), and in the SVM-GIG vs. TM-GIG comparison (**c**). Bubble color indicates the p-value of GO term; bubble size indicates the frequency of GO terms in the underlying GOA database.

We investigated 11,692, and 12,665 DGEs in the VM vs. TM (i.e., the difference between the VM and TM stages of development, denoted VM-GIG vs. TM-GIG) and SVM vs. TM comparisons (denoted SVM-GIG vs. TM-GIG) for GIG, respectively, and 790 DEGs in the VM vs. TM comparison for OB (denoted VM-OB vs. TM-OB; Supplementary Table S1). DEGs identified in each comparison were characterized by a GO enrichment analysis to identify the biological functions of these genes. Significantly enriched GO terms belonging to the biological process (BP) and molecular function (MF) categories (p < 0.05) were identified and visualized using the ReviGO tool (Fig. 1 and Supplementary Fig. S2 and Supplementary Table S2). As highlighted in Fig. 1, the GO terms for each comparison were obviously different. In the VM-OB vs. TM-OB comparison, there was enrichment for GO terms associated with sugar metabolism, including the sucrose metabolic process, aminoglycan catabolic process, and carbohydrate metabolic process in the BP category and sugar-phosphatase activity and fructose-bisphosphate aldolase (FBA) activity in the MF category (Fig. 1a and Supplementary Fig. S2a). DNA modification, methylation, sucrose metabolic process, and the regulation of gene expression, epigenetic were enriched in VM-GIG vs. TM-GIG, while DEGs in SVM-GIG vs. TM-GIG were related to many processes, including photosynthesis, light reaction, development process, starch metabolic process, carbohydrate transport, and so on. Additionally, compared with the GO terms in the BP category identified for VM-GIG vs. TM-GIG, terms related to starch metabolism were more highly enriched in the SVM-GIG vs. TM-GIG comparison (Fig. 1b,c). We concluded that sugar metabolism is an important inductive cue affecting the floral transition in both roses, OB and GIG. Additionally, DEGs related to photosynthesis and light reaction were enriched in SVM-GIG vs. TM-GIG, implying that photoperiod plays an important role in regulating the floral transition in GIG. DEGs related to DNA modification and methylation were identified in VM-GIG vs. TM-GIG, and these genes were mainly associated with vernalization and autonomous pathways.

### Functional category enrichment of stage-specific DEGs in GIG and OB

**Figure 2:**
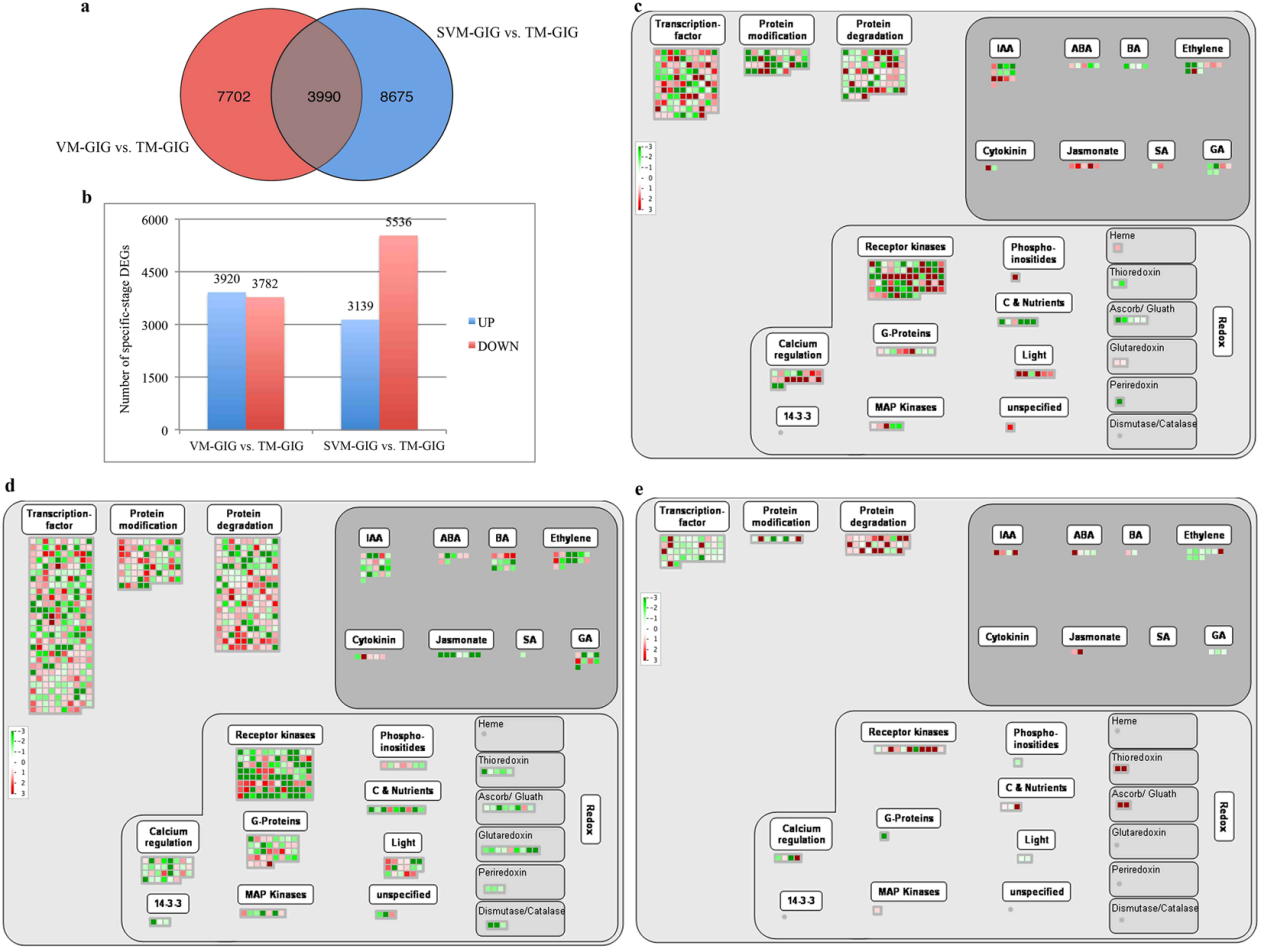
Number of DEGs and MapMan regulation overview maps in each comparison. (**a**) Number of conserved and stage-specific expression DEGs in pairwise comparisons of GIG. (**b**) Stage-specific comparison of DEGs in GIG. (**c**) MapMan regulation overview maps showing the transcript levels of stage-specific DEGs in the VM-GIG vs. TM-GIG comparison. (**d**) MapMan regulation overview maps showing the transcript levels of stage-specific DEGs in the SVM-GIG vs. TM-GIG comparison. (**e**) MapMan regulation overview maps showing the transcript levels of DEGs in the VM-OB vs. TM-OB comparison. The color indicates log_2_ value of fold changes, green color represents down-regulated transcripts, and red color represents up-regulated transcripts.

We further investigated transcriptome changes involved in the rose floral transition in response to environmental and developmental cues. We inferred that common DEGs in the VM-GIG vs. TM-GIG and SVM-GIG vs. TM-GIG comparisons were not closely associated with the floral transition. Excluding common DEGs, we identified 7,702 (up-regulated, 3,920; down-regulated, 3,782) and 8,674 (up-regulated, 3,139; down-regulated, 5,536) stage-specific DEGs in VM-GIG vs. TM-GIG and SVM-GIG vs. TM-GIG, respectively (Fig. 2a,b). MapMan was used to map these stage-specific DEGs, identify functional categories, and investigate overrepresented regulation and metabolism terms (Supplementary Table S3). Figure 2c,d shows the comprehensive regulation overview maps of stage-specific pairwise comparisons (VM-GIG vs. TM-GIG and SVM-GIG vs. TM-GIG), including hormones, RNA, protein, and so on. More stage-specific DEGs were detected in the regulation overview in SVM-GIG vs. TM-GIG than in VM-GIG vs. TM-GIG (Fig. 2c,d), revealing that the physiological and molecular properties of SVM-GIG buds were obviously different from those of VM-GIG. Regarding the IAA metabolism subcategory, more DEGs were down-regulated in SVM-GIG vs. TM-GIG than in VM-GIG vs. TM-GIG (Fig. 2c,d and Supplementary Table S3); moreover, auxin contents continued to increase from VM-GIG to SVM-GIG (Supplementary Fig. S3). Accordingly, IAA was not a key cue mediating the floral transition in GIG. DEGs associated with GA metabolism were largely down-regulated in VM-GIG vs. TM-GIG and SVM-GIG vs. TM-GIG (Fig. 2c,d), indicating that GA levels were down-regulated during the floral transition process (Supplementary Fig. S3). Therefore, GA functioned as an inhibitor regulating the rose floral transition.

Regarding RNA categories, zinc finger proteins, IDD2/7 (Unigene0016847 and Unigene0030937) were up-regulated in the SVM-GIG vs. TM-GIG comparison; these regulate the production of floral stimuli or the inhibition of floral repressors^22^. A cold-inducible zinc finger-containing glycine-rich RNA-binding protein (Unigene0028397) was up-regulated in VM-GIG vs. TM-GIG (Supplementary Table S1). Additionally, Unigene0084554 identified in VM-GIG vs. TM-GIG encodes a small glycine-rich RNA-binding protein, which is responsive to vernalization and interacts with the autonomous pathway. Importantly, two genes, Unigene0046409 and Unigene0018266, which control the vegetative to reproductive phase transition of the meristem, decreased in VM-GIG vs. TM-GIG and SVM-GIG vs. TM-GIG, respectively. It is worth noting that Unigene0001915, Unigene0002888 (*DRM1*), and Unigene0019386, associated with methylation and epigenetic regulation, were reduced in VM-GIG vs. TM-GIG (Supplementary Table S1). Similarly, Unigene0017617 and Unigene0018266, related to DNA methylation and the silencing of *FLOWER LOCUS C* (*FLC*), respectively, were down-regulated in SVM-GIG vs. TM-GIG. These changes demonstrated that vernalization plays a vital role in inducing the floral transition before the TM stage of GIG.

Further comparisons of the metabolism pathways that differ in the VM-GIG vs. TM-GIG and SVM-GIG vs. TM-GIG comparisons were performed. As shown in Fig. S4a,b, more genes in the light reactions subcategory were down-regulated in SVM-GIG vs. TM-GIG, but no DEGs related to light reactions were identified in VM-GIG vs. TM-GIG, suggesting that photoperiod affects the floral transition in GIG. Other photosynthesis genes, involved in the Calvin cycle, photorespiration, and sucrose breakdown subcategories, obviously decreased in SVM-GIG vs. TM-GIG, but did not differ between VM-GIG and TM-GIG. Furthermore, DEGs associated with starch synthesis were up-regulated in SVM-GIG vs. TM-GIG, but were down-regulated in VM-GIG vs. TM-GIG (Supplementary Fig. S4a,b). Combined with changes in the starch contents (Supplementary Fig. S3), these findings suggest that photoperiod affects photosynthesis, sugar metabolism, and further influences the rose floral transition.

More DEGs were related to major CHO metabolism, cell wall, and lipid categories in the SVM-GIG vs. TM-GIG comparison than in VM-GIG vs. TM-GIG (Supplementary Fig. S4a,b). Regarding major CHO metabolism categories, sucrose synthase (*SUS*) and beta-amylase (*BAM*) were enriched in SVM-GIG vs. TM-GIG, but not in VM-GIG vs. TM-GIG (Supplementary Table S3), indicating that the storage of energy substances, such as sugar, starch, and lipids, was less in SVM-GIG than in VM-GIG. These substances may function as energy sources, osmotic regulators, and molecular regulators of the floral transition in GIG.

To increase our understanding of the expression differences during the floral transition between OB and GIG, we also investigated functional categories that were significantly overrepresented in the VM-OB vs. TM-OB comparison. Based on comparisons of the regulation overview and metabolism overview maps for VM-GIG vs. TM-GIG, SVM-GIG vs. TM-GIG, and VM-OB vs. TM-OB (Fig. 2 and Supplementary Fig. S4), we identified potentially important regulators of the floral transition in the two roses, and the loci explaining the difference between CF and OF phenotypes. Based on the regulation overview maps (Fig. 2c,e), the expression trends of genes related to hormonal regulation was similar to the VM-GIG vs. TM-GIG comparison. For example, IAA genes were up-regulated, whereas genes related to GA decreased from VM to TM; for example, *SCL13* (c20369_g1 and Unigene0035053), a negative regulator of GA signal transduction pathway, increased during the floral transition in both roses. The DEGs c37195_g3 in VM-OB vs. TM-OB and Unigene0084554 in VM-GIG vs. TM-GIG, which not only respond to vernalization, but also interact with the autonomous flowering pathway, were identified (Supplementary Table S3). Surprisingly, genes associated with vernalization were also identified in VM-OB vs. TM-OB. For example, c21473_g1 encodes a RNA 3′-end processing factor of *FLC* silencing, and *VERNALIZATION 1* (*VRN1*), c32035_g1, is essential for the complete repression of *FLC* in vernalized plants. These results suggested that the floral transition in OB is affected by vernalization. However, OB is characterized by continuous blooming; thus, its floral transition does not appear to be controlled by vernalization. Additionally, *PERIANTHIA* (*PAN*) and *WUSCHEL* (*WUS*), which control stem cell activity in the determinate floral meristem, were also differentially expressed in OB (Supplementary Table S3).

We further compared the metabolism overviews during the floral transition in the two roses. As shown in Supplementary Fig. S4a,c, more DEGs related to reserve materials (starch and sucrose), light reactions, and glycolysis were up-regulated in OB than in GIG, suggesting that sugar metabolism plays a vital role in regulating the floral transition in OB. Regarding genes in the major CHO metabolism categories, *GRANULE-BOUND STARCH SYNTHASE 1* (*GBSS1*) and *SUS2* were identified in VM-OB vs. TM-OB (Supplementary Table S1); these important enzymes related to starch metabolism were also differentially expressed.

### Identification of candidate DEGs accounting for the floral transition in OB and GIG

**Figure 3:**
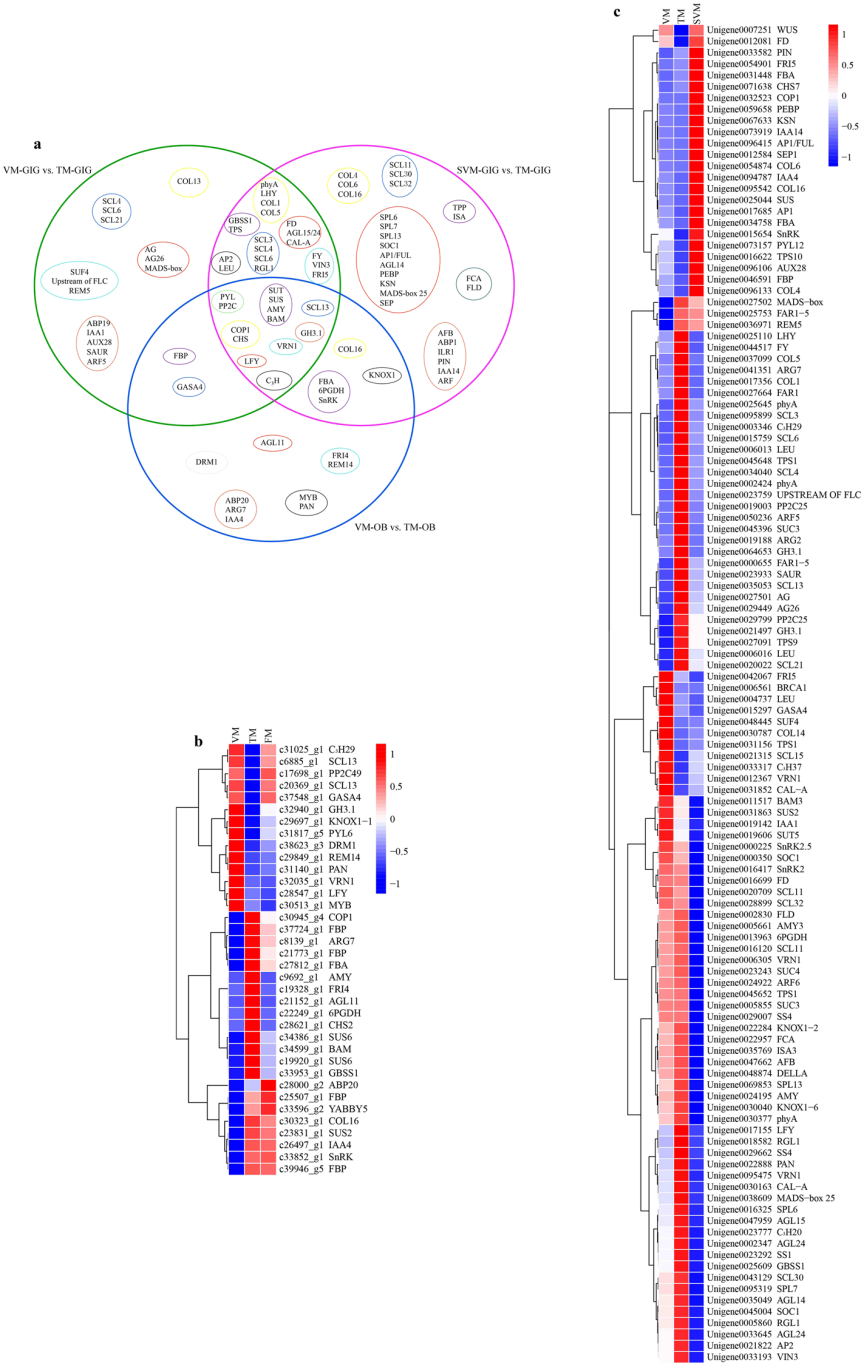
The overview of DEGs was identified between different comparisons of OB and GIG, respectively. (**a**) A Venn diagram showing the candidate floral DEGs commonly or individually belonging to each comparison of GIG and OB. Big green circle represents DEGs in the VM-GIG vs. TM-GIG comparison; big rose-red circle represents DEGs in the SVM-GIG vs. TM-GIG comparison; big blue circle stands for DEGs in the VM-OB vs. TM-OB comparison. Small yellow, cyan-blue, purple, light blue, orange-red, black, red circles indicate DEGs involved in photoperiod, vernalization, sugar metabolism, GA, auxin pathways, TFs, floral integrators, respectively. (**b**) Hierarchical clustering analysis of key floral candidate DEGs during floral transition process in OB. (**c**) Hierarchical clustering analysis of key floral candidate DEGs in different developmental stages of GIG. Data for gene expression levels were normalized by Z-score. Red and blue colors represent up- and down- regulated genes, respectively.

Based on previous studies demonstrating the molecular regulatory networks underlying floral transition^2, 21^, in the current study, we identified and summarized DEGs significantly involved in the floral transition of OB and GIG, respectively. As shown in Fig. 3, DEGs and stage-specific DEGs belonging to the VM-GIG vs. TM-GIG, SVM-GIG vs. TM-GIG, and VM-OB vs. TM-OB comparisons are displayed in a Venn diagram in Fig. 3a. Notably, sucrose transporter protein (*SUT*), *SUS*, alpha-amylase (*AMY*), and *BAM* were differentially expressed in the above three comparisons (Fig. 3a). DEGs associated with sucrose synthesis and metabolism as well as starch metabolism were together up-regulated in VM-OB vs. TM-OB, coinciding with changes in the soluble sugar and starch contents during the floral transition (Fig. 3b and Supplementary Fig. S3). Furthermore, FBA, fructose-1,6-bisphosphatase I (FBP), and 6-phosphogluconate dehydrogenase (6PGDH) jointly increased from VM-OB to TM-OB (Fig. 3b). It is noteworthy that an increased soluble sugar content is closely associated with a decreased starch content during the floral transition^23^. Thus, sugar metabolism plays a vital role in regulating the floral transition in OB. By contrast, *BAM3*, *SUS2*, trehalose-phosphate synthase (*TPS1*), and *FBP* decreased during the floral transition process in GIG, while *SUT*, *SS1*/*4*, *GBSS1*, and *AMY3* increased. *GBSS1*, *SS1*, *AMY*, and *SUS2* were up-regulated from SVM-GIG to VM-GIG (Fig. 3c). In addition, soluble sugar contents did not exhibit obvious changes between developmental stages, whereas the starch content decreased from VM-GIG to TM-GIG, and then increased again at the SVM-GIG stage (Supplementary Fig. S3). Therefore, starch metabolism had a significant influence on the floral transition in GIG. Importantly, the contents of soluble sugar and starch in the buds of OB were higher than those in the buds of GIG (Supplementary Fig. S3), indicating that sugar metabolism plays an inducing role in regulating the floral transition in OB.

DELLA proteins are central nodes that function as negative regulators in GA signaling^24^. In the study, *RGL1*, *SCL3*, *SCL4*, *SCL6*, *SCL13*, and *SCL21* were increased in VM-GIG vs. TM-GIG and SVM-GIG vs. TM-GIG, and *SCL11*, *SCL30*, and *SCL32* were only up-regulated in the SVM-GIG vs. TM-GIG comparison (Fig. 3c). Additionally, *Gibberellic Acid-stimulated in Arabidopsis 4* (*GASA4*) was down-regulated in the VM-GIG vs. TM-GIG and VM-OB vs. TM-OB comparisons (Fig. 3b,c). Simultaneously, the contents of GA_1_, GA_3_, and GA_4_ were all down-regulated during the floral transition in the two roses (Supplementary Fig. S3). Paradoxically, *SCL13* decreased from VM-OB to TM-OB (Fig. 3b). In this scenario, we speculated that *RGL1* and *SCLs* functioned as activators to induce the floral transition in GIG, and *GASA4* played a negative role in regulating the floral transition in OB.

Regarding auxin signals, auxin-binding proteins, ABP19 and ABP20, and auxin-induced proteins, ARG2 and ARG7, were up-regulated during the floral transition in the two roses (Fig. 3b,c), while auxin-responsive proteins, such as IAA1, IAA4, and IAA14 decreased. Indole-3-acetic acid-amido synthetase (GH3) facilitates the conversion of auxin to amino acids, maintaining auxin homoeostasis. *GH3*.*1* expression increased from VM-GIG to TM-GIG, but was down-regulated from VM-OB to TM-OB; in addition, auxin contents continued to increase during the floral transition in the two roses (Supplementary Fig. S3). In that respect, auxin may induce the floral transition in the two roses. However, it is worth noting that auxin contents were highest at the SVM stage of GIG (Supplementary Fig. S3), indicating that auxin was not a determinant of the regulation of the floral transition in GIG.

In the ABA signaling pathway, Pyrabactin Resistance 1-like (*PYL*) is an ABA receptor that directly inhibits protein phosphatase 2C (*PP2C*), which in turn directly represses SNF1-related protein kinases (*SnRK*)^25^. In GIG, *PYL12* and *SnRK2*/*2*.*5* decreased, but *PP2C25* increased from VM-GIG to TM-GIG (Fig. 3c). Likewise, DEGs involved in ABA signaling were down-regulated from VM-OB to TM-OB (Fig. 3b). Meanwhile, the ABA content decreased during the floral transition in the two roses (Supplementary Fig. S3), indicating that ABA functioned as an inhibitor in inducing the floral transition in the two roses.

The floral transition of temperate plants is usually mediated by seasonal cues, including photoperiod (circadian clock) and vernalization. Phytochrome A (*phyA*), *LATE ELONGATED HYPOCOTYL* (*LHY*), and *CONSTANS-LIKE* (*COL1*, *COL3*, and *COL5*), involved in photoperiod and circadian clock pathways, were up-regulated in VM-GIG vs. TM-GIG, and *phyA*, *LHY*, and *COL5* also increased from SVM-GIG to TM-GIG (Fig. 3b,c). Unexpectedly, *COL4*, *COL6*, and *COL16* were down-regulated from SVM-GIG vs. TM-GIG (Fig. 3c). During the floral transition of OB, *CONSTITUTIVE PHOTOMORPHOGENIC 1* (*COP1*), *CHALCONE SYNTHASE 2* (*CHS2*), and *COL16* were up-regulated (Fig. 3b), indicating that photoperiod plays an inducing role. While *COP1* decreased in VM-GIG vs. TM-GIG and SVM-GIG vs. TM-GIG, *CHS7* increased in VM-GIG vs. TM-GIG, but decreased in SVM-GIG vs. TM-GIG, indicating that photoperiod functions as an activator, and there was potential functional diversification (Fig. 3b,c).

Seasonal perennials require vernalization to obtain the competence to complete floral transition, even if plants return to warmer temperatures^26^. *SUPPRESSOR OF FRI4* (*SUF4*) decreased, but *REPRODUCTIVE MERISTEM 5* (*REM5*) increased from VM-GIG to TM-GIG (Fig. 3c). *VERNALIZATION INSENSITIVE 3* (*VIN3*) and *FY* were up-regulated, and *FRIGIDA 5* (*FRI5*) was down-regulated in VM-GIG vs. TM-GIG and SVM-GIG vs. TM-GIG comparisons. As shown in Fig. 3, *VERNALIZATION 1* (*VRN1*) was up-regulated in VM-GIG vs. TM-GIG and SVM-GIG vs. TM-GIG, but decreased from VM-OB to TM-OB (Fig. 3b,c). In addition, *FCA*, *FLOWERING LOCUS D* (*FLD*), and *SQUAMOSA PROMOTER BINDING PROTEIN-LIKE* (*SPL6*/*7*/*13*) were up-regulated in SVM-GIG vs. TM-GIG (Fig. 3c). Unexpectedly, *FRI4* was identified and increased from VM-OB to TM-OB (Fig. 3b). These phenomena suggested that vernalization had a significant role in regulating the floral transition of GIG, whereas its roles in OB require further experiments to examine potential functional diversification in regulating floral transition.

Environmental and developmental cues eventually converge to a series of floral integrators and floral meristem identity genes. In this study, *LFY* and MADS-box genes, including *AGL15*/*24*, *CAL-A*, and *FD* were jointly up-regulated in VM-GIG vs. TM-GIG and SVM-GIG vs. TM-GIG (Fig. 3a,c). Additionally, *MADS-box*, *AG*, and *AG26* increased from VM-GIG to TM-GIG. *SOC1*, *AGL14*, and *MADS-box 25* were up-regulated in the SVM-GIG vs. TM-GIG comparison, but *AP1*, *AP1* (*FUL*), *PEBP*, and *SEP* were down-regulated (Fig. 3a,c). Taken together, we concluded that *SOC1*, *LFY*, *AGL14*/*15*/*24*, *CAL-A*, *MADS-box 25*, *KSN*, and *SPL6*/*7* function as vital activators controlling the floral transition in GIG. Moreover, a hierarchical clustering analysis showed that these genes exhibited high co-expression (Fig. 3c). In OB, *LFY* had high expression during the floral transition, and *AGL11* increased from VM-OB to TM-OB, while *DRM1* and *MYB* decreased (Fig. 3b). It is noteworthy that a hierarchical clustering analysis revealed high co-expression patterns among *DRM1*, *REM14*, *PAN*, *VRN1*, *LFY*, and *MYB* (Fig. 3b). Hence, these DEGs played a substantial role in the regulation of the floral transition in OB.

### Verification of gene expression by RT-qPCR

**Figure 4:**
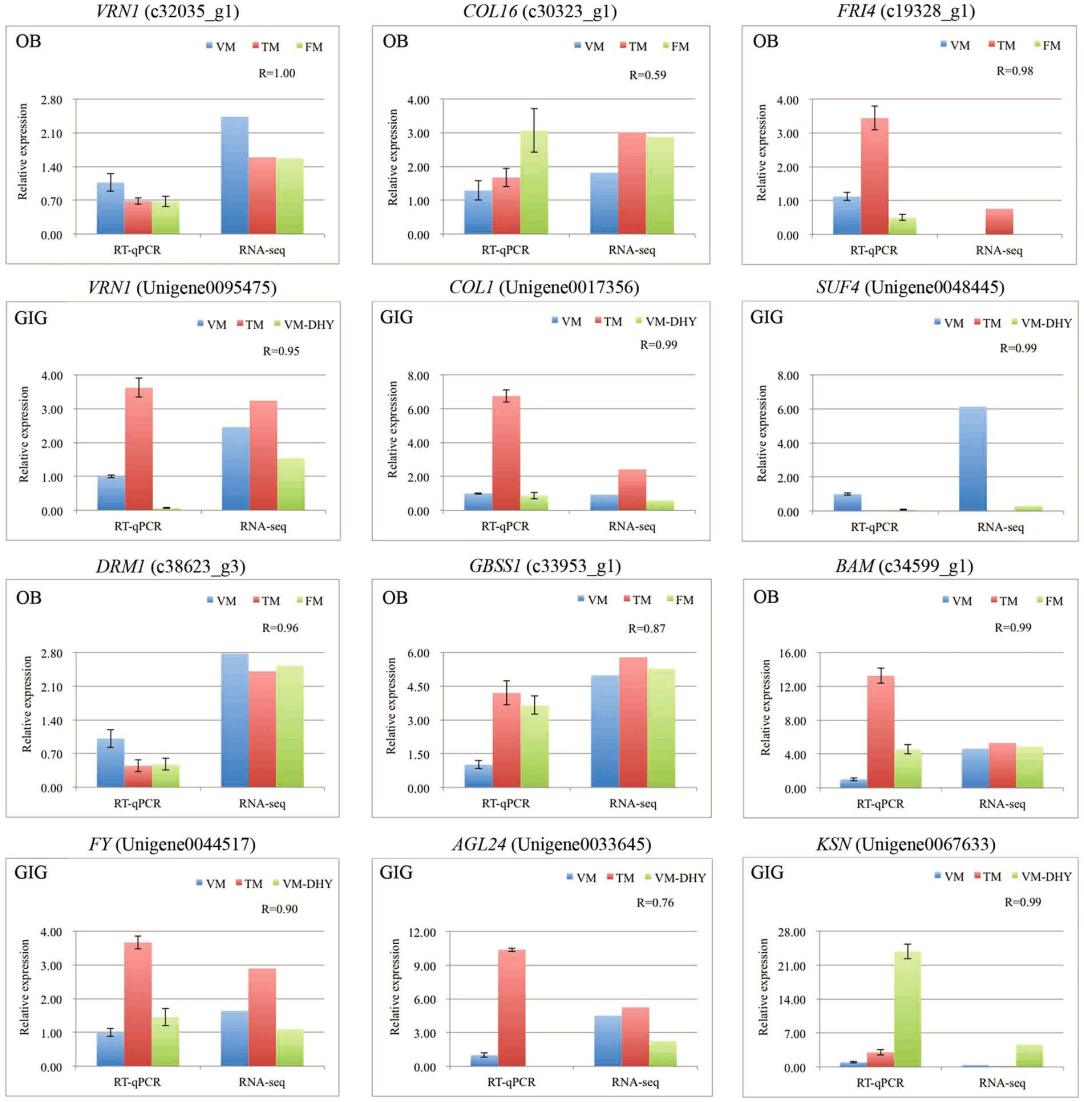
RT-qPCR validation of key candidate floral DEGs in different developmental stages of OB and GIG. The expression levels of genes revealed by RT-qPCR (left side) and RNA-seq (right side). Data from RT-qPCR were means of three replicates and bars represent SE. Data from RNA-seq were means of replicates and were normalized by Log^2^ transformed. The correlation coefficient (R) for each gene between RT-qPCR and RNA-seq data was shown and calculated by cor.test in R.

To confirm the reliability of the RNA-seq data, we selected a few key candidate floral DEGs, involved in vernalization, photoperiod, and sugar metabolism pathways, for parallel RT-qPCR-based expression analysis in OB and GIG. The expression profiles of genes obtained by RT-qPCR and RNA-seq were highly similar, confirming the accuracy of our transcriptomic analysis (Fig. 4).

## Discussion

Sugar metabolism is an important process in plant development; it not only provides an energy source, but also acts as a florigenic signal during the floral transition in *A*. *thaliana* ^27^. Starch is the key form of carbon reserve in plants, including storage starch and transitory starch, in which the dynamic polymer within transitory starch is regulated by the circadian clock and *GBSS1*, which directly promotes amylose synthesis^28^. Starch accumulation and the glycan composition change dramatically during the floral transition process and are controlled by *CO* ^23^. Moreover, increased sugar levels are concomitant with decreased starch levels during the floral transition process. The presence of *CO* in the promoter of *GBSS1* was established by ChIP experiments, indicating that *CO* directly regulates the expression of *GBSS1* ^23^. In the current study, the soluble sugar contents increased slightly during the floral transition in GIG. The starch content decreased dramatically and subsequently increased at the SVM-GIG stage (Supplementary Fig. S3). At the same time, *COL5* and *GBSS1* synchronously increased in VM-GIG vs. TM-GIG and SVM-GIG vs. TM-GIG comparisons (Fig. 3c), suggesting that starch metabolism plays a significant role in regulating the floral transition in GIG. The trends in soluble sugar and starch contents in OB were similar to those in GIG during the floral transition (Supplementary Fig. S3), and *COL16* was up-regulated from VM-OB to TM-OB (Fig. 3b). In addition to starch, trehalose-6-phosphate (T6P) is another floral stimulus that coordinates the floral transition in *A*. *thaliana* by itself or via the regulation of *miR156* or *SPL* in the shoot apical meristem^29^. Interestingly, T6P also regulates the accumulation and turnover of transitory starch^30^. By contrast, the content of soluble sugar in OB was far more greater than that in GIG (Supplementary Fig. S3), and this property may be associated with continuous flowering, an economically valuable trait, in OB.

6PGDH activity is closely related to the floral transition in *Brassica campestris* and functions as a good enzyme marker for the floral transition^31^. In this study, the expression pattern of 6 PGDH coincided with the phase change from vegetative growth to flowering in the two roses (Fig. 3b). Another enzyme, FBA, is mediated by circadian clock and plays a dominant role in regulating flowering time via sugar metabolism^32, 33^. Hierarchical clustering analysis revealed that FBA was highly correlated with *COP1*, with up-regulated expression profiles (Fig. 3b). Indeed, *COP1* mediates the control of light input to the circadian clock, involved in the floral transition of *A*. *thaliana* ^34^.

Sugar signals involve extensive interactions with hormone signals, affecting the transition from vegetative to reproductive growth^35^. Generally, *T6P* signaling is an indicator of sucrose transduction^36^. Various lines of evidence show that *T6P* represses the expression of *SnRK* when the content of sucrose exceeds a certain threshold concentration, and the *T6P*-*SnRK* module orchestrates changes in some floral genes, such as *SPL* and *miR156*, to regulate juvenility and floral signal transduction^37^. *SnRK* also participates in ABA signaling and functions as a positive regulator of ABA signaling. As shown in Supplementary Fig. S3, ABA levels and soluble sugar contents showed opposite trends during floral transition in the two roses. In GIG, *SnRK* decreased from VM-GIG to TM-GIG and had lower expression than that at SVM-GIG, but *SnRK2*/*2*.*5* exhibited the opposite trend in expression. Likewise, *SnRK2* was up-regulated from VM-OB to TM-OB. Further experiments will be needed to examine these patterns.

It has recently been demonstrated that GA operates positively and then negatively to mediate the floral transition in *A*. *thaliana*. GA initially activates the expression of *LFY*, whereas up-regulated *LFY* in turn reduces GA levels in a negative feedback loop, causing DELLA enrichment, which further promotes *AP1* expression to induce the floral transition^38^. In OB, *LFY* exhibited the highest expression at the VM stage (Fig. 3b), indicating that *LFY* expression was activated quickly, before the floral transition commences, as observed in *A*. *thaliana* ^39^. Moreover, the GA levels decreased sharply from VM-OB to TM-OB (Supplementary Fig. S3). In that respect, GA might also influence *LFY* in OB. DELLA protein acts as a negative regulator of GA. Previous studies have shown that DELLA functions as an activator in the floral transition of *Prunus avium* and *Citrus unshiu* ^40, 41^. Furthermore, DELLA could interact with *SPL* via *miR156*, and further regulate *SPL* and *MADS-box* genes in *A*. *thaliana* ^42^. In addition, sugar is able to activate *SPL* via *miR156* combined with the aging pathway^43^. Yuan and Wysocka-Diller also demonstrated that *RGL2* participates in sugar metabolism and regulates the floral transition^44^. In this study, the expression the DELLA protein RGL1 and *TPS1*/*9* corresponded with the phase-change of GIG (Fig. 3c), suggesting that RGL1 is probably involved in sugar metabolism. Furthermore, linkage between plant architecture and flowering behavior has been identified and is associated with some quantitative trait loci (QTLs). Moreover, some genes associated with GA biosynthesis and auxin signaling have been identified in the vicinity of these QTLs^45^.

After a period of vernalization, several flowering inhibitors, such as *FLC* and *FRI*, are silenced epigenetically via histone modifications^26^. *FLC* is also inhibited by the autonomous pathway, including *FY*, *FCA*, and *FLD* ^46^. In addition, *VRN1*/*REM5*, activated by vernalization, act as promoters in inducing floral transition^46^. In GIG, key genes involved in vernalization and autonomous pathways play activating roles in inducing flowering, as observed in *A*. *thaliana* ^26^, suggesting that vernalization and autonomous pathways have significant influences on the floral transition in parallel. Unexpectedly, genes associated with vernalization, including *FRI4*, *VRN1*, and *REM14*, were also identified in OB, which exhibits continuous flowering throughout the year. However, their expression trends were different from those of GIG during the floral transition (Fig. 3b,c), suggesting that vernalization is not a dominant pathway affecting the floral transition of OB or that these genes underwent functional diversification. *DRM1* is an important locus that promotes flowering; it mediates the expression of *SOC1* and *LFY* and is involved in the autonomous pathway^47^. In addition, *DRM1* is also related with apical dominance or paradormancy, and is repressed by increasing auxin and soluble sugar contents, but is activated by ABA^48, 49^. *DRM1* expression was associated with physiological changes, such as auxin, soluble sugar, and ABA (Figs 3b and S3), indicating a role in the floral transition in OB.

It has been demonstrated that the bZIP transcription factor *PAN* interacts with various developmental pathways, such as light, hormones, and meristem control systems^50^. Moreover, *PAN* can activate the expression of *AG* ^51^. As shown in Fig. 3b, *PAN*, *DRM1*, and *REM14* were highly correlated, suggesting that methylation and epigenetic regulation caused by the autonomous or vernalization pathways mediated the expression of *PAN*, and further regulated the floral transition from vegetative to reproductive growth. Additionally, a plethora of MADS-box transcription factors were identified in this study. It was noteworthy that *AGL24* was identified in GIG but not in OB, and it originated from a branch of *DAM* involved in perennial dormancy that is controlled by seasonal factors, including vernalization and photoperiod^52^. In this way, the floral transition in GIG was influenced by vernalization.

## Methods

### Plant material and experimental procedures

Samples of OB and GIG were collected from Kunming Yang Chinese Rose Gardening Co., Ltd in the Yunnan Province of China (24°45′N, 102°53′E). The vegetative meristem (VM), pre-floral meristem (TM), floral meristem (FM), and secondary vegetative meristem (SVM) stages of OB and GIG were identified by a histological analysis^53^ (Supplementary Fig. S1). Samples of each stage of OB are abbreviated VM-OB, TM-OB, FM-OB, and samples of different developmental stages in GIG are abbreviated VM-GIG, TM-GIG, FM-GIG, and SVM-GIG. The samples obtained for the VM-OB, TM-OB, and FM-OB stages were collected on December 29, 2014, January 2, 2015, and January 8, 2015, respectively, while samples of VM-OB, TM-OB, FM-OB, and SVM-GIG were obtained on December 27, 2014, January 2, 2015, January 8, 2015, and March 8, 2015, respectively. The scales and young leaves outside of buds were peeled off as soon as possible, so that the remaining tissue was closer to the shoot apical meristem. Each biological replicate contained mixed buds (~0.30 g), and each stage had three biological replicates. All samples were collected from 12:00 to 17:00, and quickly placed in liquid nitrogen and stored at −80 °C until further use. Samples for paraffin sections, RNA extraction, and hormone, soluble sugar, and starch measurements were collected at the same time.

### Measurements of soluble sugar, starch, and hormone contents

Almost 0.1 g fresh samples at different developmental stages of OB and GIG were respectively collected for the measurement of soluble sugar and starch, and at least 0.05 g for the hormone measurement. The former was measured according to the sulfuric acid-anthrone colorimetric method^54^. High-performance liquid chromatography-mass spectrometry (HPLC - MS) (AB 5500, Beijing, China) was used to identify and quantify hormones as previously described^55^.

### Transcriptome analysis

The quality and quantity of RNA was determined using the NanoPhotometer@ spectrophotometer (IMPLEN, Westlake Village, CA, USA). Approximately, 3 μg of RNA per sample was used for the construction of libraries using the NEBNext® UltraTM RNA Library Prep Kit for Illumina® (NEB, Ipswich, MA, USA) following manufacturer’s instructions. Library quality was assessed using the Agilent Bioanalyzer 2100 system. Three biological replicates were sequenced, paired-end sequencing was conducted using an Illumina HiSeq^TM^ 4000. After filtering raw reads, clean reads were *de novo* assembled using the Trinity short reads assembling program^56^, producing two transcriptome reference databases for OB and GIG, respectively. All sequence data of OB and GIG were deposited in the Genome Sequence Archive with project ID PRJCA000258 and project ID PRJCA000286, respectively. RPKM (Reads Per kb per Million reads) was used to calculate the relative expression levels^57^. Differential expression analysis of each comparison was performed using the DESeq R package (1.10.1), and the p-values correction (false discovery rate, FDR) was adjusted using the Benjamini and Hochberg method^58^. The identification of differentially expressed genes (DEGs) between two groups was evaluated with |fold change|>1.5 and FDR < 0.05.

The web-based ReviGO software (http://revigo.irb.hr) was used to identify gene ontology (GO) functional categories (p < 0.05) associated with each set of compared samples^59^. The DEGs were annotated using the Mercator web tool and then loaded into MapMan software for function enrichment analysis^60, 61^.

### Data validation by real-time quantitative PCR (RT-qPCR)

Total RNA treated with DNase (Takara, Dalian, China) was reverse transcribed to produce cDNA templates for reactions according to the manufacturer’s instructions (Takara, Dalian, China). For RT-qPCR, the reaction was carried out with a qTOWER 2.2 PCR System (Jena, Germany) and SYBR Green PCR Master Mix (Takara, Dalian, China) according to the manufacturer’s protocol. The primer sequences for RT-qPCR were designed using the PrimerQuest Tool (Supplementary Table S4), and synthesized by Sangon Biotech Co., Ltd. (Beijing, China). The *RcActin* gene was used as a reference gene^62^. Each 20 μL reaction mixture contained 2 μL cDNA template. The amplification program was as follows: 3 min at 95 °C and 40 cycles of 10 s at 95 °C and 30 s at 60 °C. Relative expression levels of candidate genes were calculated using the 2^−ΔΔCt^ method^63^. Each reaction was performed with three biological replicates. RNA-seq data were Log-transformed prior to the Pearson’s correlation evaluation. Pearson’s correlation values between RNA-seq and RT-qPCR data of selected genes were calculated using cor. test in R version 3.3.

## Electronic supplementary material

Supplementary information

Supplementary Table S1

Supplementary Table S2

Supplementary Table S3

Supplementary Table S4
