# Supplementary material for: Comparative transcriptome analysis of the floral transition in Rosa chinensis ‘Old Blush’ and R. odorata var. gigantea
Source: Sci Rep. 2017 Jul 20;7:6068. doi: 10.1038/s41598-017-05850-8 (PMC5519770; doi:10.1038/s41598-017-05850-8)
Supplement: Supplementary file 1 — Supplementary information [file 41598_2017_5850_MOESM1_ESM.pdf]

Title:

Comparative transcriptome analysis of the floral transition in *Rosa chinensis* 'Old Blush' and *R. odorata* var. gigantea

Author:

Xuelian Guo[+], Chao Yu[+], Le Luo, Huihua Wan, Yushu Li, Jia Wang, Tangren Cheng, Huitang Pan, Qixiang Zhang*

Authors' addresses:

Beijing Key Laboratory of Ornamental Plants Germplasm Innovation & Molecular Breeding, National Engineering Research Center for Floriculture, Beijing Laboratory of Urban and Rural Ecological Environment, Key Laboratory of Genetics and Breeding in Forest Trees and Ornamental Plants of Ministry of Education, School of Landscape Architecture, Beijing Forestry University, Beijing, 100083, China

[+]These authors have contributed equally to this work.

*Corresponding auther:

Qixiang Zhang

E-mail：zqxbjfu@126.com

Supplementary information

This file contains four supplementary figures and captions of four supplementary files.

Figure legends

Fig. S1. Morphological characteristics of shoot apical meristem (SAM) between different developmental stages in OB and GIG. Morphological changes of SAM were analyzed at a histological level: (A) the upper panel represents the floral transition process of OB, (B) the down panel shows the floral transition process and the secondary vegetative meristem of GIG. The morphology of SAM is narrow at the VM stage, and the SAM becomes broad and showed a pronounced conic which indicates the evidence of phase-change from the VM to TM stage. Then, the SAM sequentially differentiates sepal primordia, and turns into the FM stage. The secondary axillary buds of GIG maintain vegetative growth after flowering, which was identified at the SVM stage. Black bar: 50μm.

Fig. S2. Scatterplot of enriched GO in pairwise comparisons. The scatterplot of enriched GO terms ($p < 0.05$) in molecular function in the VM-OB vs. TM-OB comparison (A), in the VM-GIG vs. TM-GIG comparison (B), and in the SVM-GIG vs. TM-GIG comparison (C). Bubble color indicates the p-value of GO terms; bubble size indicates the frequency of GO terms in the underlying GOA database.

Fig. S3. Measurement of hormones, soluble sugar and starch contents in different developmental stages of OB and GIG, respectively. Values are means of three replicates ± SE.

Fig. S4. MapMan analysis showing differences between pairwise comparisons. (A) MapMan metabolism overview maps showing the transcript levels of stage-specific DEGs in the VM-GIG vs. TM-GIG comparison. (B) MapMan metabolism overview maps showing the transcript levels of stage-specific DEGs in the SVM-GIG vs. TM-GIG comparison. (C) MapMan metabolism overview maps showing the transcript levels of DEGs in the VM-OB vs. TM-OB comparison. The color indicates log_2 value of fold changes, green color represents down-regulated transcripts, and red color represents up-regulated transcripts.

Figure S1

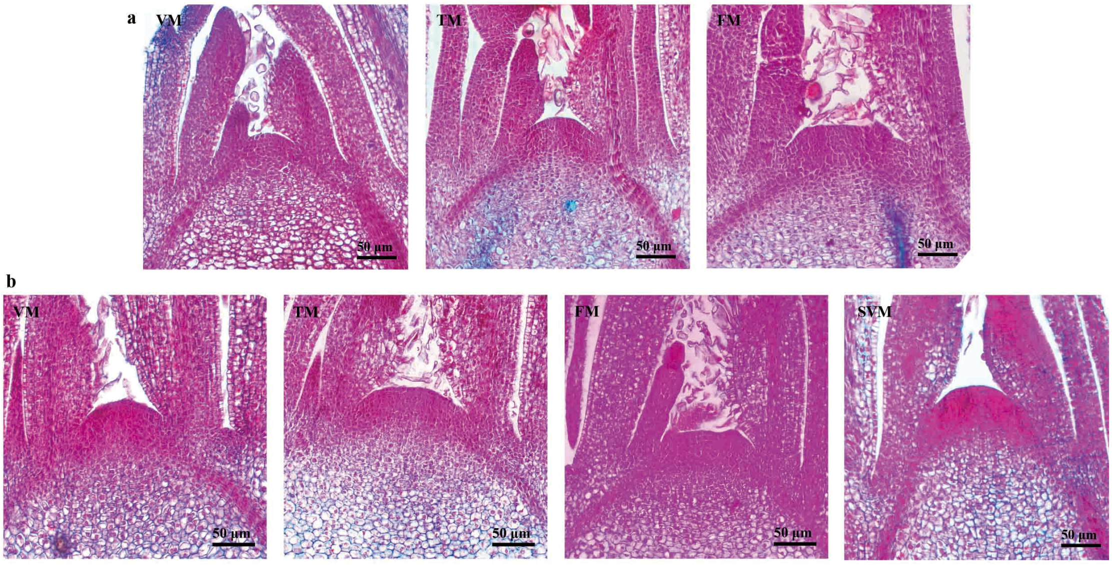

Figure S2

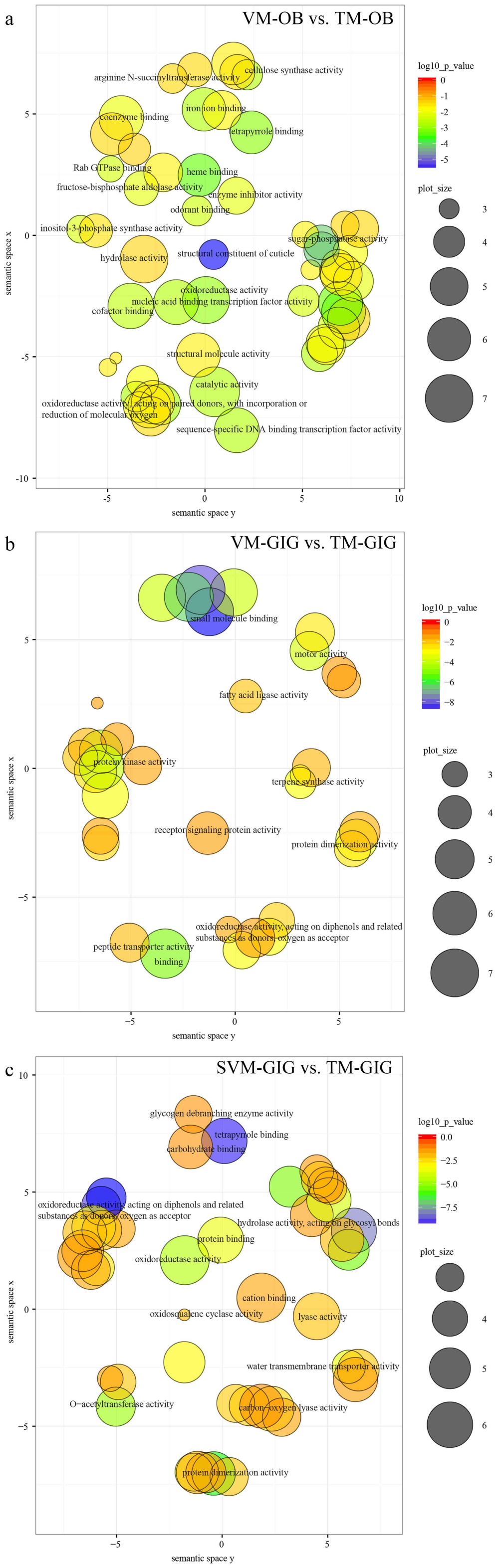
a
VM-OB vs. TM-OB
arginine N-succinyltransferase activity
cellulose synthase activity
iron ion binding
coenzyme binding
tetrapyrrole binding
Rab GTPase binding
heme binding
fructose-bisphosphate aldolase activity
enzyme inhibitor activity
odorant binding
inositol-3-phosphate synthase activity
sugar-phosphatase activity
hydrolase activity
structural constituent of cuticle
oxidoreductase activity
nucleic acid binding transcription factor activity
cofactor binding
structural molecule activity
catalytic activity
oxidoreductase activity, acting on paired donors, with incorporation or reduction of molecular oxygen
sequence-specific DNA binding transcription factor activity
semantic space x
semantic space y
log10_p_value
plot_size
b
VM-GIG vs. TM-GIG
small molecule binding
motor activity
fatty acid ligase activity
protein kinase activity
terpene synthase activity
receptor signaling protein activity
protein dimerization activity
oxidoreductase activity, acting on diphenols and related substances as donors, oxygen as acceptor
peptide transporter activity
binding
c
SVM-GIG vs. TM-GIG
glycogen debranching enzyme activity
tetrapyrrole binding
carbohydrate binding
oxidoreductase activity, acting on diphenols and related substances as donors, oxygen as acceptor
hydrolase activity, acting on glycosyl bonds
protein binding
oxidoreductase activity
cation binding
oxidosqualene cyclase activity
lyase activity
water transmembrane transporter activity
O−acetyltransferase activity
carbon−oxygen lyase activity
protein dimerization activity

Figure S3

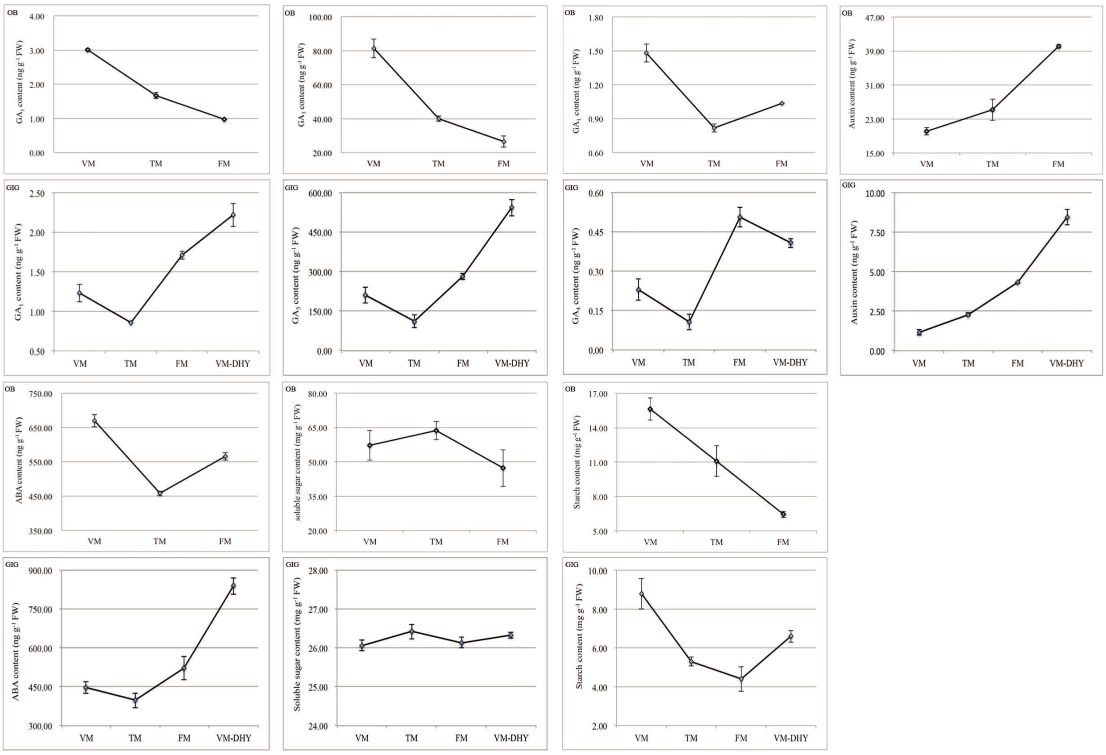
OB
GIG
VM
TM
FM
VM-DHY
Auxin content (ng g-1 FW)
ABA content (ng g-1 FW)
soluble sugar content (mg g-1 FW)
Starch content (mg g-1 FW)
Soluble sugar content (mg g-1 FW)

Figure S4

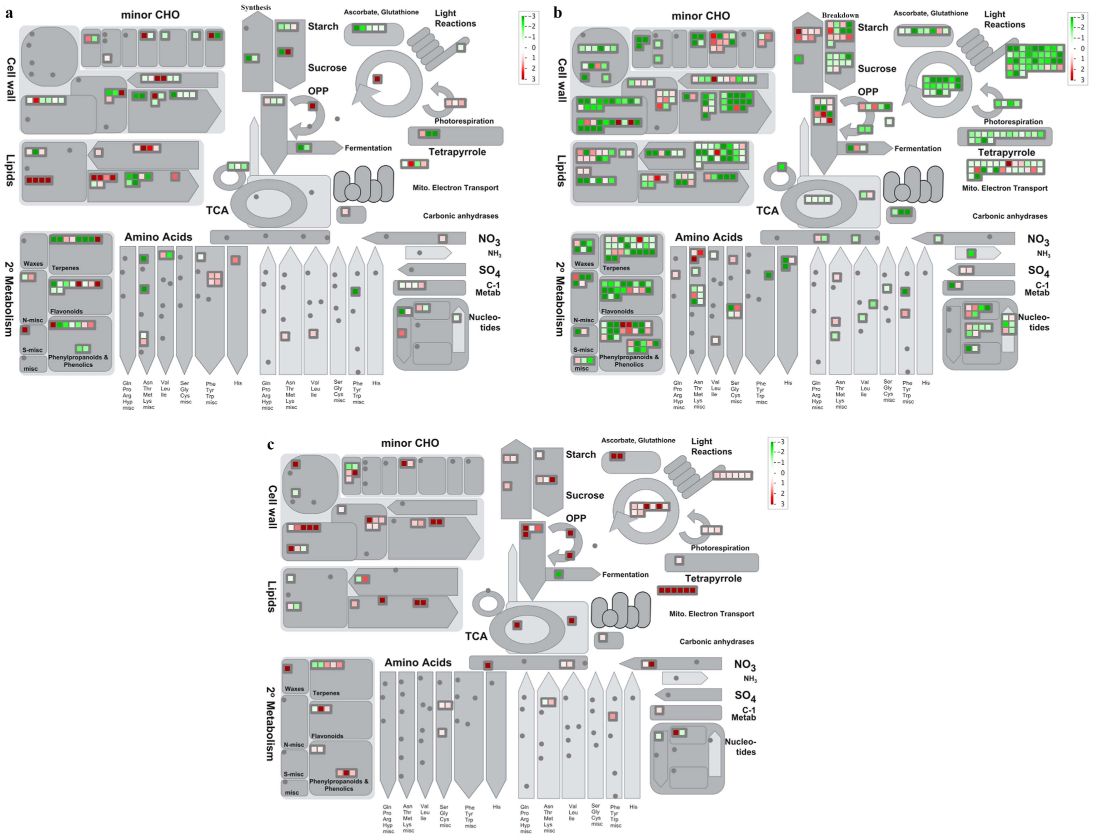

a
minor CHO
Cell wall
Lipids
Synthesis
Starch
Sucrose
OPP
Ascorbate, Glutathione
Light Reactions
Photorespiration
Fermentation
Tetrapyrrole
TCA
Mito. Electron Transport
Carbonic anhydrases
Amino Acids
NO3
NH3
SO4
C-1 Metab
Nucleo- tides
2° Metabolism
Waxes
Terpenes
Flavonoids
N-misc
S-misc
misc
Phenylpropanoids & Phenolics
Gln Pro Arg Hyp misc
Asn Thr Met Lys misc
Val Leu Ile
Ser Gly Cys misc
Phe Tyr Trp misc
His
b
Breakdown
c

Supplementary Table S1. The identification of DEGs in each comparison of OB and GIG, respectively.

Supplementary Table S2. The relevant GO terms of DEGs in each comparison, including VM-GIG vs. TM-DH, SVM-GIG vs. TM-GIG, and VM-OB vs. TM-OB respectively.

Supplementary Table S3. The annotation of DEGs between pairwise comparisons by using the Mercator software.

Supplementary Table S4. Primers used to assay gene expression by RT-qPCR.
